# Collaborative research: Accomplishments & potential

**DOI:** 10.1186/1476-069X-7-3

**Published:** 2008-01-21

**Authors:** Klea Katsouyanni

**Affiliations:** 1 Department of Hygiene, Epidemiology and Medical Statistics, University of Athens, Athens, Greece

## Abstract

Although a substantial part of scientific research is collaborative and increasing globalization will probably lead to its increase, very few studies actually investigate the advantages, disadvantages, experiences and lessons learned from collaboration. In environmental epidemiology interdisciplinary collaboration is essential and the contrasting geographical patterns in exposure and disease make multi-location projects essential. This paper is based on a presentation given at the Annual Conference of the International Society for Environmental Epidemiology, Paris 2006, and is attempting to initiate a discussion on a framework for studying collaborative research. A review of the relevant literature showed that indeed collaborative research is rising, in some countries with impressive rates. However, there are substantial differences between countries in their outlook, need and respect for collaboration. In many situations collaborative publications receive more citations than those based on national authorship. The European Union is the most important host of collaborative research, mainly driven by the European Commission through the Framework Programmes. A critical assessment of the tools and trends of collaborative networks under FP6, showed that there was a need for a critical revision, which led to changes in FP7. In conclusion, it is useful to study the characteristics of collaborative research and set targets for the future. The added value for science and for the researchers involved may be assessed. The motivation for collaboration could be increased in the more developed countries. Particular ways to increase the efficiency and interaction in interdisciplinary and intercultural collaboration may be developed. We can work towards "the principles of collaborative research" in Environmental Epidemiology.

## Introduction

Collaborative research may be conceptualized as a research effort done by research groups from different disciplines (interdisciplinary collaboration), either belonging to the same country (national) or to more than one country (international) or it may be a parallel research effort by groups from different countries applying the same protocol across various locations or a combination of the above. Collaborative research, mainly from complementary groups within one country, happened in the past. However, increasing globalization, characterized by the facility in communications and exchange of information, is already followed and is likely to continue by an impressive increase in the number, size and diversity of collaborative research projects. This phenomenon is widely recognized; however its characteristics, benefits, drawbacks and efficiency are seldom the objectives of systematic investigation [1].

In Environmental Epidemiology specifically, there are additional reasons for which collaboration in research is very important and there is accumulated experience on these: one is collaboration for better exposure assessment (eg. in air pollution or meteorological factors) between scientists working in exposure assessment and epidemiologists and the second reason is that through collaborative efforts researchers are able to study larger contrasts in exposure and health profiles between various geographical areas [2]. In spite of this reality, in scientific journals with environmental epidemiology topics very little is found on formal discussion and assessment of collaborative research.

This paper, which is based on a talk delivered at the Annual Conference of the International Society for Environmental Epidemiology, Paris 2006, is attempting to initiate a discussion on a framework for studying collaborative research.

## Background

Some study of collaborations in research for all disciplines, with the use of specific indices, has been undertaken within the Bibliometrics/Scientometrics literature [3]. This work has mainly focused on international co-authorship of scientific publications, which is hypothesized to reflect to a certain extent international collaboration. It is evident that this approach, although providing some valuable information on the issue, cannot reflect all aspects of collaboration. In this section, a brief review of the extent and trends in collaborative research is presented and a reference on the European experience guided by the European Commission (E.C.), probably the only systematic effort to promote international collaboration in research, is made.

### Trends in collaborative research

We hypothesize that the facilitation of communications and the dispersion of information characterizing our times, certainly gives more incentives for collaboration in research. But is this indeed the case?

In a study of co-authorship, in which a paper is defined as international if the co-authors come from at least 2 different countries, a comparison between papers published in 1985/86 and 1995/96 indeed revealed a strong increase in the number of international papers [4].

In Table 1 the share of international publications as well as the percent increase between 1985/86 and 1995/96 for various countries can be seen. It appears that collaboration, at least as reflected in the frequency of international co-authorship in published papers, is indeed increasing. The country with the biggest share of international co-authorships is Thailand (64%). In the list of the first 20 countries we see 12 European. The UK ranks 40^th^, the US 47^th ^and Japan last, with 27%, 18% and 14% share of international papers respectively. For all countries, without exception, an increase in international papers has been observed between 1985/6 and 1995/6. However, the largest increases have been observed in Eastern European countries (e.g. Romania 200%, Czech Rep 158%) where major social changes were observed during this period. It would be good to have the same documentation for the more recent years, however this was not found in the published literature. Glanzel [3], one of the most active workers in Bibliometrics, mentions that although the increasing trend continued after 1995, the rate of increase was lower.

**Table 1:** Share of international co-publications in 1985/86 and 1995/96 for selected countries among the 50 most active (All fields, ranked in decreasing order; adapted from [4])

Rank & Country	Share % 95/96 – 85/86 (% increase)	Rank & Country	Share % 95/96 – 85/86 (% increase)
1. Thailand	64 – 47 (36)	12. Slovakia	44 – 19 (132)
2. Hungary	50 – 27 (85)	13. Denmark	43 – 24 (79)
3. Portugal	50 – 38 (32)	15. Mexico	43 – 30 (43)
4. Czech Rep	49 – 19 (158)	16. Austria	43 – 24 (79)
5. Switzerland	48 – 32 (50)	17. Brazil	42 – 27 (56)
6. Poland	46 – 20 (130)	18. Bulgaria	40 – 21 (90)
7. Chile	45 – 26 (73)	20. Norway	40 – 23 (74)
8. Belgium	45 – 28 (61)	40. U.K.	27 – 14 (93)
9. Venezuela	45 – 31 (45)	47. U.S.A.	18 – 10 (80)
10. Romania	45 – 15 (200)	50. Japan	14 – 7 (100)

**Figure 1:**
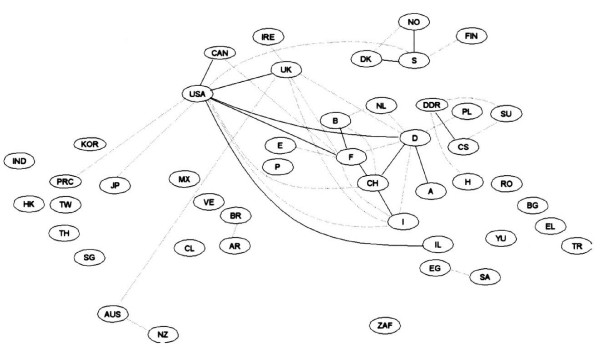
International co-authorship map for the 50 most active countries in all scientific fields combined in 1985/86. Salton's measure dotted line >1.5%; solid line >2.5%; reprinted from [4] with permission no 1827631363292.

**Figure 2:**
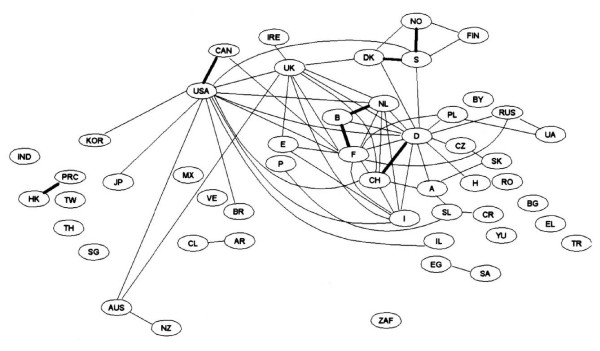
International co-authorship map for the 50 most active countries in all scientific fields combined in 1995/96. Salton's measure solid line >2.5%; thick line >5%; reprinted from [4] with permission no 1827631363292.

In Figure 1 we see the links between different countries, as expressed in joint paper authorships for all scientific fields combined [4]. The links are quantified with the use of Salton's measure [5] which may be seen as the proportion of collaborative publications between two countries over the total number of internationally co-authored publications in both countries. Therefore an increasing value in Salton's measure reflects an increase in bilateral collaboration over and above the general increase in internationally co-authored papers. A dotted line in Figure 1 signifies a Salton's measure >1.5% and solid line >2.5%. We observe links between the US and Canada, the US and European countries, important inter-European links, links within Scandinavian countries etc. In Figure 2 we can see how impressively the links have increased. The solid lines are now much more numerous and the thick lines represent Salton's measure >5%, which did not exist before. The links existing in 1985/6 have been strengthened and new links (eg. between Hong Kong and China, between Australia and New Zealand) have been established.

### Recognition and visibility

It has been investigated whether the product of a collaboration gains more "visibility" compared to research confined within a single country. One way of assessing this is through the study of the number of citations received by a paper compared to the average expected for a specific journal.

An analysis based on selected countries, including Germany, Japan, Denmark, New Zealand, Russia, India, Hungary and Greece, showed that international papers have a significantly increased "attractivity" (ie a larger number of citations) compared to single country (domestic) ones and this fact is more pronounced for the less developed countries [4].

**Figure 3:**
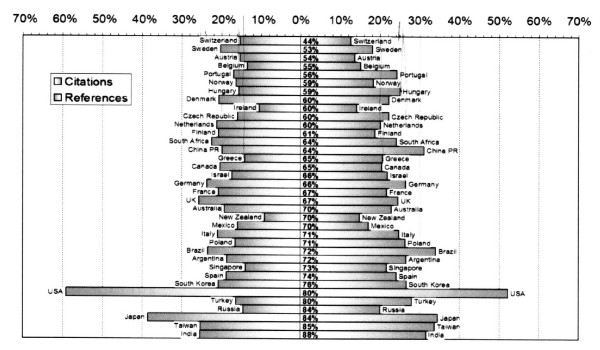
Reference (left), citation (right) and authorship (axis) domesticities in Biomedical research reprinted from [6] with permission no 1827640162250.

An interesting study investigated the ways in which authors from different countries cite domestic (i.e. from their own country) and international literature [6]. If reference lists in published papers merely reflected the proportion by which papers from a specific country were represented in the international literature, then the inclusion of domestic papers from a specific country in a reference list and the proportion of citations received for these papers would coincide. The study reveals significant differences (Figure 3).

In Table 2 some examples are shown from publications in the biomedical field: Thus e.g. US authors cite US papers by a higher percentage (59%) than are cited by others (52%). The same pattern may be observed for Japanese authors, whilst UK and German authors refer to their national papers by the same proportion as they are cited by others. The opposite pattern is observed e.g. for Greek authors who cite their domestic papers by a smaller percentage (15%) than are cited by others (20%). This latter pattern is also observed in Hungary.

**Table 2:** Selected examples of percentage of domestic papers, percentage of domestic papers in reference lists and percentage of citations received in the field of biomedical research. Adapted from [6].

Country	% domestic papers	% they cite domestic	% they are cited by others
USA	80	59	52
Japan	86	39	35
UK	67	26	25
Germany	66	24	26
Greece	65	15	20
Hungary	59	16	26

A limited search, focusing on environmental epidemiology (Table 3), showed that the percentage of national papers in the US Journal "Epidemiology", for the years 1999–2000, found using the keywords "air pollution or particles and mortality", was 71% whilst during the same period in the "European Respiratory Journal" they were 53%. Most of the international papers in the European Respiratory Journal were in fact inter-European. In these two journals there appear to be no differences in the number of citations received by National or International papers (Table 3). To investigate these issues more thoroughly, a more extensive study has to be undertaken. However, it appears that in a large country, with many researchers and centralized data bases, the added value of collaborative research cannot readily be perceived.

**Table 3:** Percentage of national and international papers in a U.S. and a European journal on "air pollution or particles and mortality" published in 1999–2000.

Type of paper	Epidemiology N papers (%) N citations	Eur Respir J N papers (%) N citations
National	17 (71) 53	10 (53) 18
(US)	9 (38) 68	1 (5) 6
(other)	8 (33) 37	9 (48) 18
International	7 (29) 54	9 (48) 23
Inter-European	1 (4) 16	6 (32) 25

### Collaborative research in Europe

Europe is currently the most important host of collaborative research. The E.C. has been the main driver of collaborative research in Europe, through its successive Framework Programmes (FP). The E.C. promotes the position that the effectiveness of research depends critically on the strength of networking between research partners and across research disciplines [7]. Experience has accumulated, and now the E.C. is at the first stages of the implementation of FP7. The E.C. has asked for an evaluation of FP6 and in Table 4 some aspects of this evaluation may be seen [8].

**Table 4:** Networking through funded projects: a comparison of the European Commission's Framework Programmes (FP)*

	FP3	FP4	FP5	FP6
Number of organizations in funded projects	6291	5335	8026	3351
Number of funded projects	2131	1743	2786	374
Average number of organizations per project	7.1	7.1	7.2	15.1
Average number of projects per organization	2.4	2.3	2.4	1.7

* from the final report "Evaluation of Networks of collaboration among participants in IST research and their evolution to collaborations in the European Research Area" (ERAnets [8])

An alarming characteristic of FP6 is illustrated in Table 4: The number of organizations involved in FP6 has decreased by a factor of 2.5 compared to FP5! A smaller number of organizations may mean a disadvantage for smaller and less developed countries and for smaller institutions. This may lead to the formation of an "elite" and the exclusion of new, fresh ideas from interacting and fertilizing "the establishment". Further, the number of projects has been divided by 7.5., as FP6 favored fewer and larger projects. This fact may deprive some countries and organizations of ever coordinating a project. Larger projects and more partners per project often lead to less "personal" management. Often professionals are employed to manage the projects leading to decreased personal contacts between researchers. Meetings tend to be too large and look more like Conferences, with formal presentations instead of direct interaction through discussion between participants. As a result, participants sometimes find it hard to identify with the collaborative project. Literature from other disciplines supports the previous points. This Cowan and Jonard [9] note that "Weak ties and shifting networks are preferable to strong ties and stable networks when the goal of an activity is to encourage new knowledge creation. The geographic distribution may be encouraging innovation at a rate not experienced in close collaboration or stable networks" and Wagner [10] concludes that "...collaborators with-a-difference may be more likely to challenge or perhaps complement the outlook and capabilities of others (and) maybe more likely to result in innovative research and intriguing outcomes (compared to stable networks)."

The E.C. during assessment of FP6 and preparation of FP7 has critically reviewed and adjusted its practices of choosing tools and funding projects and it is calling for smaller consortia under FP7.

## Discussion

From the review of the literature and the existing situation in the EU, it may be inferred that indeed there is an increase in collaborative research and that selective links are established between counties which share a similar culture. It can also be seen that collaboration may be provoked and its patterns dictated by specific policies such as those implemented by the E.C. Further it seems that international papers receive more feasibility that national ones and the difference is more pronounced for authors who work in less developed countries.

However, important aspects of collaborative research have not been addressed, to our knowledge, in a systematic way. Thus several advantages of collaborative research have been mentioned in the literature [1] and various other key issues may be discussed. A tentative list and related open questions are presented below.

### 1. Better access to expertise, equipment and resources

This advantage may be pivotal for interdisciplinary research and to what extent it is in fact realized remains to be investigated. An important related aspect is to what extent there is transfer of know-how from one country to another, thus contributing to the development of expertise and research capabilities in countries which lack a specific experience. A systematic evaluation of past collaborative research will illustrate these aspects. Indeed, it seems sometimes particularly difficult to establish effective communication between groups working in different disciplines. The scientists who belong and work in one discipline often take several views for granted and can easily use familiar terminology to communicate. The barriers between fields are not so easy to break. Even after several collaborative projects, it is found that the various disciplines have in fact worked in parallel and did not really interact.

### 2. Better access to funds

This is not necessarily true for countries with large national research funding schemes. For the sake of research, there should be access to national as well as international project funding and the optimum balance should be kept. However, in some countries, even developed ones, national funding is limited and distributed in non-transparent ways. This aspect could also be the objective of investigation. The results of such an investigation could lead policies on research, such as for example the E.C. policy.

### 3. More prestige and visibility

As seen above, there is some evidence that collaborative projects produce papers which are more widely sited compared to national ones published in the same journal. There is no evidence provided in the literature about the probability to publish papers in higher impact journals if these are collaborative or co-authored by a more "prestigious" center. Also the evidence hints to the possibility that the relative advantage is not true for countries with very developed research, like the U.S.

### 4. Better efficiency and productivity, more speed or, on the contrary, more bureaucracy?

What is the balance between collaborative research and the single researcher or single group small project, which may be more innovative and certainly more flexible? We should realize that in spite of the many advantages of collaboration it involves more bureaucratic procedures. The reasons and ways through which these procedures develop and may affect the collaboration to a greater or lesser extent has not been systematically investigated.

### 5. Taking advantage of the varied environmental conditions

In environmental epidemiology, one of the most important assets of collaborative research is taking advantage of the large variability in environmental conditions across the World. This is already implemented and is likely to happen more and more often in the future. This is an area where working on principles to guide collaborations and to ensure the mutual benefit of all the involved groups is of outstanding importance.

### 6. There may be more focus and less mistakes made (since there is internal "evaluation")

This advantage is less obvious than it may seem and whether there is internal evaluation or not has to be studied.

### 7. A political decision?

In the European Union there is a specific policy to encourage collaboration in research and particular advantages are seen in this procedure. A result of the collaboration is increased homogenization of the countries which form the European Union. Similar advantages may be seen more globally. More collaboration in research promotes equity, understanding and peace. Ultimately, it is a political decision.

## Conclusions & suggestions for the future

The lack of evaluation of past experience of research collaborations does not help with future plans and setting targets. It will be useful to study the characteristics and dynamics of past collaborative research, addressing the specific issues raised above.

It is proposed here that we should expand our attempts to answer the questions: Do we want to encourage collaborative research? With what specific characteristics and for which specific purposes?

In some situations (e.g. in the European Union) a positive answer has been given, although the characteristics and the objectives may be periodically revised, but in others there has been limited reflection on this topic.

It is not to be expected that collaborative research will progress spontaneously to the desired directions. Specific policies will encourage and orientate collaborations. Within this context, it may also be considered to change the ways is which scientists are evaluated: shifting from the predominance of using the quantity of papers to more qualitative criteria and finding ways for their meaningful implementation. Particular ways to increase the efficiency and interaction in interdisciplinary and intercultural collaboration may be developed.

Within Environmental Epidemiology, where collaborative research is very important, we can work collectively towards "the principles of collaborative research".
